# Lipid findings from the Diabetes Education to Lower Insulin, Sugars, and Hunger (DELISH) Study

**DOI:** 10.1186/s12986-019-0383-2

**Published:** 2019-08-27

**Authors:** Ashley E. Mason, Laura R. Saslow, Patricia J. Moran, Sarah Kim, Hiba Abousleiman, Robert Richler, Samantha Schleicher, Veronica M. Goldman, Alison Hartman, Cindy Leung, Wendy Hartogensis, Frederick M. Hecht

**Affiliations:** 1 UCSF Department of Psychiatry, Center for Health and Community, San Francisco, CA USA; 2 UCSF Osher Center for Integrative Medicine, 1545 Divisadero Street, Suite 301, San Francisco, CA 94115 USA; 3 Department of Health Behavioral and Biological Sciences, The University of Michigan, School of Nursing, Ann Arbor, MI USA; 4 UCSF Division of Endocrinology, Diabetes and Metabolism, Department of Medicine, San Francisco General Hospital, San Francisco, CA USA; 5 University of Maryland, School of Medicine, Annapolis, MD USA; 6 Department of Psychology, Drexel University, College of Arts and Sciences, Philadelphia, PA USA; 7 Department of Nutritional Sciences, University of Michigan, School of Public Health, Ann Arbor, MI USA

**Keywords:** Diabetes, Low-carbohydrate diet, LDL-C cholesterol, LDL-P cholesterol

## Abstract

**Background:**

A carbohydrate-restricted (CR) diet can improve glycemic control in people with type 2 diabetes mellitus (T2DM). There are concerns, however, that the high dietary fat content of CR diets can increase low-density lipoprotein cholesterol (LDL-C), thus increasing cardiovascular disease (CVD) risk. Quantifying CVD risk associated with changes in LDL-C in the context of CR diets is complicated by the fact that LDL-C reflects heterogeneous lipids. For example, small LDL particle number (sLDL-P) is more closely associated with CVD risk than is total LDL-C, and CR diets tend to decrease the proportion of sLDL-C in LDL-C, which standard lipid measures do not indicate. Advanced lipoprotein assays, such as nuclear magnetic resonance (NMR) testing, can subfractionate lipoproteins by size and density and may better depict the effects of CR diets on CVD risk.

**Methods:**

Adults (*N* = 58) with T2DM (*n* = 37 women; baseline HbA1c ≥ 6.5%) completed a 6-month group-based CR diet intervention. We obtained a standard lipid panel, advanced lipoprotein assays (NMR testing), and two 24-h diet recalls at baseline and post-intervention (6 months). Participants also completed home-based blood ketone testing (a biological index of dietary adherence) during the final five weeks of the intervention.

**Results:**

From baseline to post-intervention, participants had increased mean HDL-C, decreased triglycerides and triglyceride/HDL ratio, decreased mean sLDL-P, and increased LDL size, which reflect reductions in CVD risk (*p*s < 0.05). Participants did not have statistically significant changes in total cholesterol, non-HDL-C cholesterol, LDL-P, or HDL-P. Twelve participants (23.1%) had a ≥ 5% increase in sLDL-P. Exploratory analyses revealed that participants with sLDL-P increases of ≥ 5% reported larger increases in servings of red meat than participants without sLDL-P increases of ≥ 5% (+ 0.69 vs − 0.29 servings; *p* = 0.033). Changes in saturated fat intake were not associated with changes in sLDL-P.

**Conclusions:**

Among most participants, we observed changes in several lipid measures consistent with decreased CVD risk. Approximately one in four participants evidenced increases in sLDL-P. Further research should clarify whether individuals with increased sLDL-P after implementing a CR diet can reverse observed increases by limiting red meat consumption.

**Trial registration:**

ClinicalTrials.gov, NCT03207711, Registered 6/11/2017. Retrospectively registered.

## Introduction

Carbohydrate-restricted (CR) diets are a non-pharmacological intervention approach that can improve glycemic control and reduce medication requirements in individuals with type 2 diabetes mellitus (T2DM) [1]. CR diets may be more effective in improving glycemic control than low-fat diets [2–4]. CR diets for T2DM treatment thus warrant further investigation, as the global diabetes epidemic may affect more than 400 million individuals worldwide [5].

Although CR diets have shown promise in improving glycemic control for individuals with T2DM, researchers have raised concerns regarding adverse effects of CR diets on blood lipids, particularly low-density lipoprotein (LDL-C) cholesterol levels [6]. Some data suggest that CR diets, especially those that restrict carbohydrates such that the body produces a low level of ketones (“ketogenic diets”), can increase LDL-C levels [7], though other data suggest that these increases may normalize over time (i.e., 15 months [8]).

Elevated LDL-C has been associated with increased CVD in epidemiological studies [9–11], and has also been associated with greater consumption of red meat [12, 13] and saturated fat [14, 15]. Greater red meat consumption [16, 17] and, somewhat questionably, greater saturated fat consumption [18–20] have also been associated with increased CVD risk in epidemiological studies. LDL-C has thus been proposed as a mechanism linking red meat and saturated fat consumption with CVD [21]. As a result, some dietary guidelines have advocated reducing red meat and saturated fat consumption to reduce CVD risk [22, 23]. CR diets commonly involve increased red meat and saturated fat consumption, which has raised concerns about the effects of CR diets on CVD risk.

One of the challenges of assessing the impact of CR diets on CVD risk is that LDL-C, as measured in standard lipid panels, does not yield information about heterogeneous sub-types that differ in their metabolic origins and pathogenic roles. Advanced lipoprotein assays (nuclear magnetic resonance [NMR] lipid measures) can separate the total LDL cholesterol into different sub-types with differential associations with CVD risk. For example, small dense LDL particles (sLDL-P) appear to be particularly linked to greater CVD risk [24]. LDL particle number (LDL-P) and LDL particle size better predict CVD risk than the standard LDL-C measure [25]. Given similar total LDL-C levels, greater levels of sLDL-P result in greater LDL-P. LDL-P may be particularly helpful in improving accuracy of CVD risk prediction among individuals with T2DM [26, 27]. Thus, advanced lipoprotein testing that goes beyond the standard lipid measure of LDL-C may better reflect the effects of dietary interventions on CVD risk.

Recent research suggests that although CR diets may increase total LDL cholesterol in some individuals, CR diets tend to decrease the LDL particle number and shift LDL particle subtypes away from sLDL-P [28]. Although few studies have employed advanced lipid testing to clarify the effects of CR diets on blood lipids, available data suggest limited adverse effects of most CR diets on blood lipids [29]. Thus, in the context of CR diets, increased LDL-C may be offset in its effects on CVD risk through shifts toward more favorable LDL subtypes and/or metrics. To better define the effects of a CR diet on lipids in T2DM, we examined changes in both a standard lipid panel and an advanced lipoprotein assay among individuals in a trial testing behavioral approaches to increase adherence to a CR diet for individuals with T2DM.

## Methods

### Study design

We randomized participants in a 1:1 ratio to one of two group-based 3-month in-person nutritional and behavioral intervention arms and followed participants for a 3-month post-intervention period (6 months of study participation total). Both arms received CR diet instruction, but one arm also received mindful eating training. We recruited three waves of approximately 20 participants each (*N* = 58 total). Participants provided blood specimens (LabCorp, Inc. location of choice) and also completed other assessments not included in these analyses. The University of California, San Francisco (UCSF) Institutional Review Board approved all study procedures. All participants provided written informed consent prior to enrollment. See Mason and colleagues [30] for further trial details.

### Participants

We recruited participants from several sources. We sent letters about the study to patients with T2DM who have been seen in UCSF clinics, posted flyers in the community, and posted ads on social media websites such as Facebook, Nextdoor, and Craigslist. Participants were 18 years of age or older; had a diagnosis of T2DM (6.5% ≤ HbA1c < 12.0% confirmed by blood test at screening); were not lactating, < 6 months postpartum, pregnant, or planning to become pregnant in the next 6 months; had not had bariatric surgery in the prior 18 months; reported no substance misuse, medical issue, or other health conditions that would make it difficult to participate; did not follow vegan or vegetarian dietary patterns; owned and used a smartphone; were willing to complete the study regardless of randomization arm; and endorsed experiencing food cravings several times per week. See Mason and colleagues [30] for more detailed inclusion and exclusion criteria related to behavioral measures.

### Interventions

Participants in both arms completed 12 weekly in-person sessions followed by 3 monthly in-person maintenance sessions, for a total of 15 sessions over 6 months.

#### Carbohydrate-restricted (CR) diet instruction

Participants in both arms received identical instruction in following a CR diet, similar to that in our previously published work, from the same group leader [31, 32]. We instructed participants to reduce their carbohydrate intake to between 20 and 35 non-fiber grams of carbohydrates per day (with the goal of remaining under 50 non-fiber grams per day), to eat an adequate amount of protein (as described by the Institute of Medicine) [33], and to eat fat to satiety. We advocated a gradual transition towards this CR diet over the first three weeks of the intervention by instructing participants to change their breakfasts and snacks in the first week, lunches in the second week, and dinners in the third week. After about 4 weeks, participants were instructed to be fully implementing the prescribed CR diet. The specific content of participants’ diets varied, but generally included green leafy and other non-starchy vegetables, avocados, nuts, seeds, oils (except trans fats), butter, fish, poultry, meats, eggs, cheese, and low-carbohydrate fruits such as strawberries and blackberries. We instructed participants to avoid sugar-sweetened foods (e.g., desserts such as cakes, cookies, and ice cream), sugar-sweetened beverages, naturally sweet foods (e.g., tropical fruits), and starchy foods (e.g., foods made with grain-based flours such as bread, pasta, tortillas, breakfast cereals, and pastas, as well as potatoes and rice). We also noted evidence of the association of red-meat consumption with some cancers, and suggested approaches to implementing a CR diet without relying on red meat. A board-certified internal medicine physician who has transitioned patients with T2DM onto CR diets in her private practice led the CR diet instruction.

#### Mindful eating training

Participants randomized to the mindful eating arm received mindful eating training in addition to the CR diet classes. We delivered mindful eating training in the form of a smartphone application that contained 28 video modules and instructed participants to watch two or three modules per week. These modules focused on mindful eating topics including breaking the automatic eating habit loop and coping with food cravings without eating. Participants who received the mindful eating training attended weekly hour-long sessions with a mindful eating teacher who answered questions and led discussions about module content (see [30] for details). As all participants received identical CR diet instruction, which we hypothesized to be the primary driver of changes in lipid profiles among participants, we combined both intervention arms for the current analyses. We confirmed that changes in lipid measures did not differ by intervention group.

### Measures

We collected demographic information, diabetes-related information, anthropometric data, standard lipid panels, and NMR lipoprotein assays from all participants. We assessed participants’ HbA1c, fasting blood glucose, and fasting blood insulin using standard procedures at a Clinical Laboratory Improvement Amendments certified clinical laboratory (LabCorp, Inc.). Participants self-reported exogenous insulin use and years since T2DM diagnosis. We assessed participants’ weight using a digital scale and height using a wall-mounted stadiometer (Doran Scales, Inc., Model DS1100). We computed body mass index (BMI) from these assessments.

#### Lipid assays

We used the NMR LipoProfile III assay (LabCorp, Inc.) to assess sLDL-P, LDL-P, average LDL particle size, HDL-P, and the lipoprotein insulin resistance (LP-IR) index. This assay produces results from lipoprotein particle analysis using nuclear magnetic resonance (NMR) spectroscopy (400 MHz) [34]. We examined the following measures collected or computed from a standard lipid panel (LabCorp, Inc.): triglycerides, LDL-C, HDL-C, non-HDL cholesterol, and triglycerides/HDL ratio.

#### Blood ketones

In week 4, we provided participants with a home-based blood ketone monitoring device (Precision Xtra® System; Abbott Diabetes Care, Alameda, California) and ketone strips. Participants used these supplies to measure β-hydroxybutyrate (BOHB) [35] in their blood. We taught participants how to use the monitoring device in person and asked them to measure their blood ketones before dinner, two to three times a week, on alternating days. Participants reported ketone measurements via an online survey on a weekly basis. The ketone monitoring devices store data, which staff checked at every weekly class (starting in week 5) and every monthly class (starting at month 4) to confirm self-reported measurements. We defined our target BOHB level as between 0.3 and 3.0 mmol/L. Although the level of carbohydrate restriction needed to achieve ≥0.3 BOHB varies between individuals, most individuals need to restrict to fewer than 50 g per day of non-fiber carbohydrate to achieve this blood ketone range [36]. For the current analyses, we used ketone data from the final five weeks of the 6-month study period.

#### Dietary intake

We collected 24-h recalls from participants via telephone using the University of Minnesota’s Nutrition Data System for Research (NDSR, Nutrition Coordinating Center, University of Minnesota) software. This is a widely used dietary analysis program that can assess a wide variety of foods. Although some recommendations suggest that a single 24-h assessment at each timepoint is adequate for longitudinal assessments in a clinical trial, we collected two assessments at each timepoint (baseline, 3 months, and 6 months) to increase accuracy (one on a weekday and one on a weekend day) [37]. Trained research assistants conducted the dietary recalls, which we conducted without prior notification (to avoid changes in diet on the reporting day). We averaged data across the two days of recalls. We conservatively defined implausible energy intakes as < 500 or ≥ 5000 kcal/day and excluded these recalls from analysis. We adjusted foods for total energy using the residual method and the population average total energy intake at each timepoint [38]. We adjusted nutrients for total energy intake using the density method. We used the following foods and nutrients in analyses: red meat (beef, veal, pork, and lamb), processed meat including red processed meat (ham, bacon, sausage, hot dog, and cold cuts) and white processed meat (turkey sausages and hot dogs, and poultry cold cuts), red and processed meats combined, and percentage of daily calories from saturated fat.

### Analytic plan

First, we computed descriptive statistics as means and standard deviations or counts and percentages. Second, we computed two-sided paired-samples *t*-tests comparing lipids measures at baseline and at post-intervention. We report means at each timepoint, mean changes from baseline, 95% confidence intervals, and *p*-values. We used *p* < 0.05 as our criterion for statistical significance.

Third, we assessed the sensitivity and specificity of standard lipid panel measures (LDL-C, triglyceride/HDL-C ratio, and non-HDL cholesterol [25, 39]) to detect participants with increases of 5% or greater (≥5%) in sLDL-P, and used standard equations for these measures [40]. We set an increase of ≥ 5% as a value that exceeded the typical coefficient of variation of the sLDL-P assay [41], which therefore likely reflected a real and meaningful increase (rather than assay variation).

Fourth, we compared participants with, versus without, increases in sLDL-P of ≥5% in terms of dietary adherence, dietary intake, and exogenous insulin use [41]. Because (1) the group with increases of ≥5% in sLDL-P included fewer than 20 participants, (2) the number of participants was not balanced between groups, and (3) we were comparing values that were not normally distributed, we used non-parametric tests (Mann-Whitney U-test and Spearman rank correlation) for these comparisons [42]. We investigated dietary adherence as indexed by blood ketone measures collected during the final 5 weeks of the study. We considered “adherent” to be at least two-thirds of assessments of BOHB values being ≥0.3 (we required a minimum of three measurements to compute adherence), and “non-adherent” to be fewer. We operationalized exogenous insulin use as “use” versus “non-use” of any type of insulin at 6 months. We examined changes in dietary intake variables from baseline to 6 months.

Finally, we assessed whether there were differences in the proportion of participants with and without increases in sLDL-P based on whether they were using statins or other lipid-lowering agents at baseline using Fisher’s Exact Test.

## Results

### Participant characteristics and data Descriptives

We enrolled 58 participants. On average, participants were 58.7 years of age and had been diagnosed with T2DM for 8.7 years. Most met criteria for overweight or obesity, with an average BMI of 32.2 (Table 1). Fifty percent of participants reported race/ethnicities other than non-Hispanic white, and 63.8% were female. At baseline, 8 (21.6%) female participants were pre-menopausal and 29 (78.4%) female participants were menopausal. At baseline, participants consumed an average of 205.03 (SD = 97.52) grams carbohydrates [41.68% (SD = 10.18%) of dietary calories]. More specifically, at baseline, participants consumed an average of 184.97 (SD = 91.63) grams non-fiber carbohydrates [38.40% (SD = 9.92%) of dietary calories]. After implementation of the CR diet, at 6 months, participants consumed an average of 93.58 (SD = 94.86) grams carbohydrates [19.49% (SD = 11.74%) of dietary calories]. More specifically, at 6 months, participants consumed an average of 77.43 (SD = 90.07) grams non-fiber carbohydrates [16.93% (SD = 11.49%) of dietary calories].

**Table 1:** Participant characteristics

Characteristic	*N* = 58
Age, years; M (SD)	58.7 (10.9)
Gender: Female; %	63.8
Race/Ethnicity:	
White; %	50.0
Black; %	10.3
Latino/Hispanic; %	10.3
Asian/Pacific Islander; %	20.7
Other; %	8.6
Years with T2DM; M (SD)	8.7 (7.2)
BMI, kg/m^2^; M (SD)	32.2 (6.3)
Weight, kg; M (SD)	92.0 (21.1)
HbA1c, %; M (SD)	7.7 (1.3)
Fasting Glucose (Plasma)^a^, mg/dL; M (SD)	153 (46)
Fasting Insulin^*^, μIU/mL; M (SD)	17.7 (11.8)

*Note*. T2DM = type 2 diabetes mellitus; BMI = body mass index; HbA1_c_ = glycosylated hemoglobin A1c

^a^We omitted one observation from each of fasting glucose and insulin as these were not fasting specimens

**Fig. 1:**
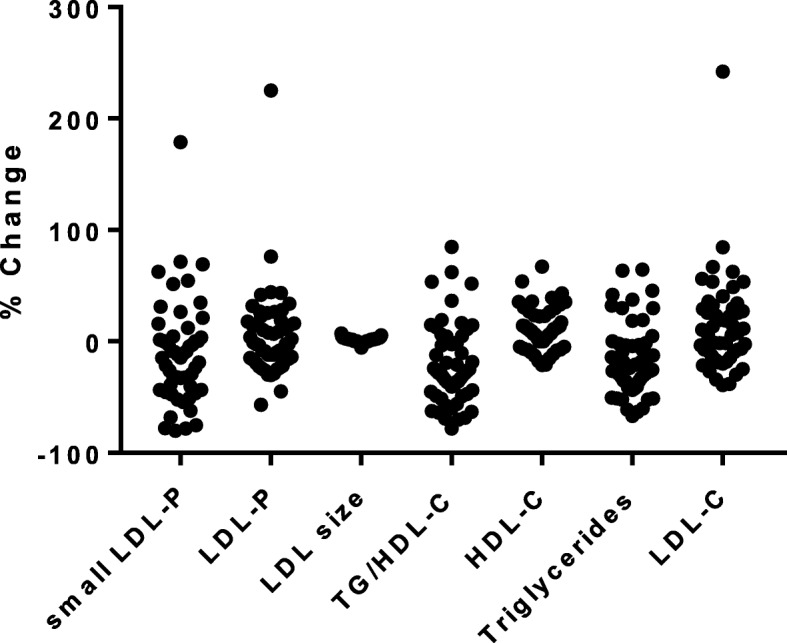
Note. Figure depicts percent change from pre- to post-intervention in lipid measures

Three participants dropped out of the study, leaving *n* = 55 who completed the intervention. An additional two participants provided non-fasting blood samples (one participant at baseline, one participant at 6 months). The former participant did not provide a sample at 6 months and was therefore not included in analyses involving change in lipid measures. We omitted some of the retained non-fasted participant’s data from analyses; specifically, we omitted changes in triglycerides and glucose (as well as baseline insulin value reported in Table 1) as these measures are impacted by fasting status [43, 44]. Inclusion of this participant did not, however, change the patterns of directionality or statistical significance of results. Laboratory error caused one participant to be missing a standard lipid panel at baseline and another participant to be missing a standard lipid panel at 6 months. On average, participants provided 8.0 (SD = 3.8) blood ketone measurements over the final five weeks of the study period. Six of the 52 (11.5%) participants in primary analyses reported in Table 2 did not provide sufficient ketone measurements (in one case, zero samples due to fingerstick aversion) for blood ketone analyses. Overall, two participants who completed the intervention were missing the 6-month 24-h diet recall data: One was missing due to travel, and other was missing due to implausible energy intake. An additional two participants (who we included in analyses of 6-month 24-h diet recall data) did not contribute baseline 24-h diet recall data: One was missing due to implausible energy intake, and the other due to missing data. See tables for sample sizes for each analysis.

**Table 2:** Change in standard lipid and NMR LipoProfile (LabCorp) from baseline to 6 months

Source of lipid measure	Lipid measure units	Mean (SD) baseline	Mean (SD) 6 months	Mean (SD) Δ from baseline	N with Δ data	95% CI [LB, UB]	*P*
Standard	LDL-C mg/dL	92.86 (38.56)	99.89 (36.51)	7.03 (29.93)	53^a^	[−1.22, 15.28]	.093
Standard	HDL-C mg/dL	52.79 (16.95)	56.49 (16.97)	3.70 (8.54)	53^a^	[1.34, 6.05]	.0027
Standard	Cholesterol, Total mg/dL	176.96 (43.23)	179.83 (44.14)	2.87 (34.30)	53^a^	[−6.59, 12.32]	.55
Standard	Triglyceride mg/dL	154.08 (78.25)	117.63 (53.57)	−36.44 (59.64)	52^a,b^	[−53.05, −19.84]	.0001
Standard	Triglyceride/HDL-C ratio	3.49 (2.59)	2.38 (1.52)	−1.11 (1.70)	52^a,b^	[−1.58, −0.64]	<.0001
Standard	Non-HDL-C cholesterol mg/dL	124.17 (41.92)	123.34 (37.84)	−0.83 (33.22)	53^a^	[−9.99, 8.33]	.86
NMR	sLDL-P nmol/L	610.58 (314.92)	493.95 (261.38)	−116.64 (255.70)	55	[−185.76, −47.51]	.0013
NMR	LDL-P nmol/L	1253.04 (430.16)	1246.60 (404.76)	−6.44 (359.33)	55	[−103.58, 90.70]	.89
NMR	LDL Size nm	20.58 (0.61)	20.82 (0.59)	0.24 (0.46)	55	[0.12, 0.37]	.0003
NMR	HDL-P (Total) μm/L	33.62 (7.07)	34.17 (6.54)	0.56 (4.53)	55	[−0.67, 1.78]	.36

*Note*. *LDL-C* = calculated low-density lipoprotein cholesterol, *HDL-C* = calculated high-density lipoprotein cholesterol, Non-HDL-C cholesterol computed as (total cholesterol – HDL-C), *sLDL-P* = small low-density lipoprotein number, *LDL-P* = low-density lipoprotein number, *LDL size* = low-density lipoprotein size; HDL-P (total) = total high-density lipoprotein number

^a^Analysis omits one participant who was missing a baseline standard lipid panel and one participant who did not provide a standard lipid panel at 6 months (*n* = 2 omitted)

^b^Analysis omits participant who provided a non-fasting sample at baseline (*n* = 1 omitted). The pattern and statistical significance of results remains if we retain the non-fasted participant

*t*-test *p*-value is two-sided. LB = Lower bound, UB=Upper bound

### Changes in lipid measures

#### Changes in standard lipid panel measures

From pre- to post-intervention, we observed statistically significant increases in HDL-C and statistically significant decreases in triglycerides and the triglyceride/HDL-C ratio on standard lipid panel testing (Table 2). These changes reflect favorable changes in CVD risk. Although total cholesterol increased an average of 7 mg/dL, this change did not reach statistical significance. There was no evidence of meaningful change in non-HDL-C cholesterol.

#### Changes in NMR lipoprotein assay measures

From pre- to post-intervention, we observed statistically significant decreases in sLDL-P and statistically significant increases in LDL size (Table 2). These changes reflect favorable changes in CVD risk. There were not statistically significant changes in LDL-P and HDL-P.

### Sensitivity and specificity of standard lipid panel measures to detect changes in sLDL-P

Of the 52 participants who provided fasting samples at baseline and 6 months, 29 (55.8%) experienced LDL-C increases of ≥5%, and 12 (23.1%) experienced changes in sLDL-P of ≥5%. Overall, standard lipid panel measures tended to achieve low sensitivity and specificity to identify participants with or without increases in sLDL-P of ≥5%, respectively (Table 3).

**Table 3:** Sensitivity and specificity of standard lipid panel measures to identify individuals at greater or lesser risk for worsened sLDL-P

Lipid measure (5% Δ)	≥5% Δ sLDL-P Sensitivity % [TP/(TP + FN)]; [95% CI]	<5% Δ sLDL-P Specificity % [(TN/(TN + FP)]; [95% CI]
LDL-C	58.3% (7/12); [27.7, 84.8]	45.0% (18/40); [29.3, 61.5]
Triglyceride/HDL-C Ratio	6/12 (50.0%); [21.1, 78.9]	85.0% (34/40); [70.2, 94.3]
Non-HDL-C Cholesterol	58.3% (7/12); [27.7, 84.8]	60.0% (24/40); [43.3, 75.1]

*Note*. Δ = Change from baseline to 6 months computed as (6 months minus baseline); *TP* = true positive, *FN* = false negative, *TN* = true negative, *FP* = false positive

### Subgroup analyses: increases (5% or greater) in sLDL-P

We next compared participants with and without ≥5% increases in sLDL-P (Table 4) to assess possible factors associated with these increases. We did not find statistically significant differences across these groups in saturated fat intake at 6 months or in changes in saturated fat intake from baseline to 6 months. Participants with ≥5% increases in sLDL-P tended to increase their red meat consumption (median change, + 0.69 servings per day), while those without such an sLDL-P change decreased their red meat consumption (median change, − 0.29 servings per day). This difference was statistically significant between these groups (*p* = 0.033, Mann-Whitney U; Table 4). There was no significant difference in processed meat consumption between these groups.

**Table 4:** Twenty-four diet recall measures of saturated fat, red meat, and processed meat intakes at 6 months and change from baseline across participants with and without sLDL-P increases of 5% or greater

Dietary measure	≥5% Δ sLDL-P Median (IQR) *n* = 11	<5% Δ sLDL-P Median (IQR) *n* = 40–42^^^	*Z-*value (Wilcoxon)	*p*-value	Median (IQR) Diff
Value at 6 months					
Saturated fat density	17.81 (14.95 to 25.40)	19.33 (16.75 to 25.20) *n* = 42	−0.790	.43	1.68 (−4.10 to 8.12)
Red and processed meat, servings/day	2.84 (1.57 to 5.24)	2.11 (1.13 to 3.76) *n* = 42	0.965	.33	−0.77 (−2.92 to 1.36)
Red meat, servings/day	2.14 (0.29 to 2.93)	0.44 (0.15 to 2.27) *n* = 42	1.382	.17	−0.72 (−2.42 to 0.56)
Processed meat, servings/day	0.72 (0.23 to 2.93)	1.12 (0.29 to 1.76) *n* = 42	0.066	.95	−0.02 (−1.30 to 1.00)
6-month change					
Saturated fat density	6.73 (3.93 to 11.75)	5.63 (2.04 to 14.41) *n* = 40	0.160	.87	−0.57 (−7.02 to 8.87)
Red and processed meat, servings/day	1.22 (−0.01 to 3.28)	0.26 (−1.63 to 1.70) *n* = 40	1.626	.10	−1.29 (−3.90 to 0.80)
Red meat, servings/day	0.69 (−0.26 to 2.06)	−0.29 (−1.28 to 0.74) *n* = 40	2.130	.033	−1.15 (−3.18 to 0.17)
Processed meat, servings/day	0.42 (−0.24 to 1.88)	0.35 (−0.53 to 1.41) *n* = 40	0.275	.78	−0.20 (−1.59 to 1.19)

*Note.* Change from baseline to 6 months computed as (6 months – baseline). ^^^ Variation in sample size between 40 and 42 is due to missing data: One participant had baseline diet recall data omitted to due to an implausible energy intake, and another had missing baseline diet recall data (see Analytic Plan and Results sections). Red meat, red and processed meat, and processed meat variables were energy-adjusted (see Methods)

To further examine associations between changes in dietary intake (of saturated fat and red meat) and change in sLDL-P, we assessed whether there was evidence of dose-response associations (using Spearman rank correlations; Table 5). There were no statistically significant associations between sLDL-P and changes in saturated fat intake. Notably, there was a statistically significant positive correlation (rho = 0.615, *p*=0.04) between increases in red meat consumption and increases in sLDL-P in the group with ≥5% increases in sLDL-P. In contrast, there was little evidence of a correlation between red meat consumption and sLDL-P in the remaining participants (rho=-0.066, *p*=0.69). We did not observe any statistically significant correlations between processed meat consumption and sLDL-P.

**Table 5:** Spearman rank correlations of change in small LDL-P with change in dietary measure from baseline to 6 months

Diet measure	Spearman rho	*p*-value	95% CI around rho
Participants with ≥5% increase in small LDL-P at 6 months (*n* = 11)			
Saturated fat, % calories	−0.200	.55	(−0.714, 0.454)
Red meat	0.615	.044	(0.024, 0.887)
Processed meat	0.123	.72	(−0.515, 0.673)
Participants with <5% increase in small LDL-P at 6 months (*n* = 40)			
Saturated fat, % calories	0.000	1.00	(−0.311, 0.312)
Red meat	−0.066	.69	(−0.370, 0.251)
Processed meat	−0.012	.94	(−0.323, 0.300)

*Note*. Red meat and processed meat variables are energy-adjusted (see Methods)

There were no statistically significant differences in dietary adherence as assessed using blood ketone measurements between those with (*M* = 44.6% adherent, SD = 38.3%, *n* = 10) and those without (*M* = 60.0% adherent, SD = 32.7%, *n* = 36) ≥5% increase in sLDL-P. Of participants with ≥5% increases in sLDL-P at 6 months who provided sufficient blood ketone measurements (*n* = 10), 30.0% were in ketosis at more than two-thirds (66.6%) of measurements at 6 months (95% CI: 6.7 to 65.2%). Of participants without ≥5% increases in sLDL-P (*n* = 36), 47.2% were in ketosis at more than two-thirds of measurementsat 6 months (95%CI: 30.4 to 64.5%). We had insufficient ketone measurements (two or fewer measurements at 6 months) for 16.7% (2/12) of participants with ≥5% increases in sLDL-P, and for 10.0% (4/40) of participants without ≥5% increases in sLDL-P. Of the 12 participants with sLDL-P increases of 5% or greater, 1 (8.3%) was using exogenous insulin. Of the 40 participants without such increases, 5 (12.5%) were using exogenous insulin. This difference was not statistically significant (Fisher’s Exact *p* = 1.00).

We also assessed the association between statins and other lipid-lowering agents with changes in sLDL-P. Sixty-seven percent of participants with and without ≥5% increases in sLDL-P were using lipid-lowering agents at baseline. One of these participants (who did not have a ≥5% increase in sLDL-P) was taking fenofibriate, and the remainder were taking statins. At 6 months, among participants without a ≥5% increase in sLDL-P, five (17%) had decreased or stopped taking lipid-lowering agents (all changes were in statins). In the group with a ≥5% increase in sLDL-P, three (37%) decreased or stopped statins (*p* = 0.33, Fisher’s exact test). Two participants in the group without a ≥5% increase in sLDL-P increased their statin dose, compared to none of the participants with a ≥5% increase in sLDL-P (*p* = 1.00).

## Discussion

In these analyses, we sought to clarify the impact of a carbohydrate-restricted (CR) diet on both standard lipid panel measures and advanced lipoprotein assay measures in people with T2DM. Previous data have shown that relative to a standard LDL-C measure, advanced lipoprotein assay measures may provide metrics that more accurately predict CVD risk [25]. Overall, our data suggested that implementing a CR diet in the context of T2DM is associated with lipid profile changes that correlate with lower CVD risk. Overall, we observed statistically significant increases in HDL and LDL size, and statistically significant decreases in triglycerides, the triglyceride/HDL-C ratio, and sLDL-P. In contrast, more than half of participants (55.8%) had increases in LDL-C. Advanced lipoprotein assay measures indicated that 23.1% [12] participants had an increase in sLDL-P of 5% or greater. Taken together, our data suggest that most people implementing a CR diet experience improved lipid profiles, but a minority may not, and may instead have increases in lipid parameters associated with CVD risk. Whereas most participants in our sample evidenced increases in the standard lipid panel measure of LDL-C, the advanced lipoprotein assay measures suggested that these increases did not accurately represent the effects of the CR diet on lipid-related CVD risk.

A variety of mechanisms may explain patterns of change in LDL subclass concentrations in the context of a CR diet. Our data suggest that CR diet composition may impact these patterns, and add to previous research showing that red meat (particularly beef) consumption may impact lipoproteins in the context of carbohydrate restriction [45]. Genetic factors, which we did not assess in this trial, may represent another factor that may influence these patterns. Furthermore, there may be an interaction between diet composition and genetic predispositions that dictates patterns of change in LDL subtypes. In our data, we found evidence that increasing red meat consumption was associated with increases in sLDL-P, though increasing saturated fat was not associated with these increases. These findings are consistent with other data showing improvements in several advanced lipoprotein measures in CR diets regardless of saturated fat content (e.g., CR diets with lower and higher levels of saturated fat content), relative to higher carbohydrate diets [46]. In a subset of our participants with increased sLDL-P, there was evidence of a monotonic (dose-response) association between changes in red meat consumption and changes in sLDL-P. Evidence for a dose-response association between red meat consumption and sLDL-P was weak in the participants without increased sLDL-P. Though very preliminary, these data support a model in which some people experience increased sLDL-P with increased red meat consumption during a CR diet, whereas others do not. Further research should replicate this finding, and, if reflective of a true process, identify potential genetic or other factors responsible for this sensitivity to red meat consumption. In addition, further research should identify what aspects of red meat consumption influence the increases in sLDL-P we observed. Though there has been concern about the effects of saturated fat intake on lipid-related CVD risk, we did not find evidence that saturated fat intake or increases in saturated fat intake were associated with increases in sLDL-P. This suggests that our observed association between red meat consumption and sLDL-P may be related to other factors, such as the precise types of fat in red meat, aspects of protein components, or other factors. We also assessed whether there was an association between adherence to carbohydrate restriction (using blood ketones as a biological marker of dietary adherence) and lipid measures and did not find this index of dietary adherence to be associated with lipid changes.

Although these data should be considered preliminary, they may hold potential implications for clinical practice. First, our data suggest that it may be useful to use advanced lipoprotein assay measures to assess the effects of a CR diet on more precise lipid measures, and to consider using these measures to adjust diet composition. In particular, assessing changes in LDL particle number and sLDL-P may be helpful in assessing the potential effects of a CR diet composition on an individual’s CVD risk, and therefore helpful in fine-tuning diet recommendations. We believe that there are several challenges in making clinical practice recommendations based upon advanced lipoprotein assays in the CR diet context. For example, availability of different advanced lipid testing assays differs by practice setting, and results have not been fully standardized across assays. This study did not compare assays, and existing data are limited in regard to whether particular versions of advanced lipoprotein assays should be recommended. In practice, clinicians may currently need to rely on assays available at a single laboratory. The implications of advanced lipoprotein assay testing for dietary recommendations would benefit from further confirmation. Reducing red meat consumption *might* be advised for people with elevated sLDL-P. In the absence of clinical trial data supporting such a practice, however, this remains a hypothesis to be tested rather than a practice that can be clearly recommended. Our data suggest that practitioners consider the *potential* impacts of increasing red meat consumption on CVD risk when suggesting dietary regimens, such as CR diets. Importantly, most of our study participants *reduced* rather than increased red meat consumption, demonstrating the feasibility of implementing a CR diet without increasing red meat consumption.

Our results differ from those reported by Chiu, et al., which found that increased small LDL particles correlated with increased saturated fat consumption in a crossover study design [47]. Several differences in study design may account for at least some of these discrepant findings. First, we used a different advanced lioprotein assayp (NMR LipoProfile) that is widely accessible but, tends to differ somewhat in results from the methods used by Chiu and colleagues [48]. Second, Chiu and colleagues’ sample was primarily male (85%), whereas ours was primarily female (64%). Third, the sources of saturated fats in the Chiu study were primarily dairy fats, whereas the sources of saturated fat may have been more variable in our study. Fourth, our CR diet prescribed a substantially smaller carbohydrate intake, and it is possible that carbohydrate intake influences the effects of saturated fat consumption on LDL subclasses.

We are aware of one other clinical trial that has examined LDL subclasses in the context of a CR diet in T2DM [29]. Data from that study revealed a similar pattern to that observed in our data. On average, data from both trials indicated significant increases in HDL and LDL size, and statistically significant decreases in triglycerides, the triglyceride/HDL-C ratio, and sLDL-P. Of note, both trials also found increases in LDL-C; however, these increases were not statistically significant in our trial. Thus, findings from our trial replicate earlier findings suggesting that CR diets may improve lipid parameters for many people with T2DM.

### Limitations

An important limitation is that our data did not allow us to examine large particle LDL sub-classes in detail. Although large LDL (relative to small LDL) may generally be associated with lower CVD risk, some forms of large LDL may have significant atherogenic potential [24]. In our results, we note that although sLDL-P decreased on average, the total LDL-P number did not decrease as much as sLDL-P. This indicates that on average, larger LDL-P increased. At a minimum, we emphasize caution about drawing too much reassurance from the finding of declines in sLDL-P. Furthermore, as in most diet patterns studied among people with T2DM, there are relatively few long-term data about the long-term impact of CR diets [49]. One study that followed patients with T2DM for 44 months did not find evidence of negative cardiovascular effects [50].

This study should be interpreted cautiously for other reasons. Our sample was small, and these findings require replication using larger samples with adequate power. Although promising, advanced lipid measures represent surrogate markers of CVD risk. A controlled trial with clinical endpoints would clarify the extent to which a CR diet impacts CVD risk, though this would require significant time and resources.

Although this study used the widely available NMR Lipoprofile, such testing is limited because it lacks standardization and comparability of information provided by other forms of advanced lipoprotein assays [51]. For example, after finding that different advanced lipid measurement methods yielded differential non-HDL-P values, Delatour and colleagues [48] recently called for standardizing these methods by use of a common commutable calibrator to improve cross-platform comparability.

A final limitation is that we used data from a clinical trial of a nutritional intervention in which participants were free to choose different foods and monitored their own carbohydrate intake. Although this represents a real-world test of what occurs when restricting carbohydrate intake, our participants' exact nutritional intake was much more variable than that in a controlled feeding study.

## Conclusions

These analyses, in tandem with those reported following a similar CR protocol [29], provide some evidence that for most people with T2DM, a CR diet is associated with favorable changes in lipid profiles (in this case, over a 6-month period). These data further support the application of caution in the interpretation of conventional standard lipid panel measure results (i.e., LDL-C) among people with T2DM who follow a CR diet and suggest possible advantages of advanced lipoprotein assays to monitor effects of CR diets on lipid-related CVD risk factors. Some people with T2DM who begin a CR diet appear to have increases in LDL subtypes associated with CVD risk (i.e., sLDL-P). For these individuals, red meat consumption may play a meaningful role in increases in sLDL-P. Future work to standardize advanced lipoprotein assay methods may facilitate the use of these methods in clinical practice. Finally, additional research should extend these findings to clinical endpoints so as to quantify actual clinical risk associated with CR diets for individuals with T2DM (i.e., CVD events).

## Data Availability

The datasets used for these analyses are available from the corresponding author on reasonable request.

## Abbreviations

ALT: Advanced lipoprotein testing
BMI: Body mass index
CR: Carbohydrate-restricted
CVD: Cardiovascular disease
HbA1_c_: glycosylated hemoglobin A1c
HDL: High density lipoprotein
HDL-C: High density lipoprotein cholesterol
LDL: Low density lipoprotein
LDL-C: Low density lipoprotein cholesterol
LDL-P: Low density lipoprotein particle
LP-IR: Lipoprotein insulin resistance index
NMR: Nuclear magnetic resonance
sLDL-P: Small LDL-P number
T2DM: Type 2 diabetes mellitus
