# Correction to: Lipid findings from the Diabetes Education to Lower Insulin, Sugars, and Hunger (DELISH) Study

**DOI:** 10.1186/s12986-019-0416-x

**Published:** 2019-12-30

**Authors:** Ashley E. Mason, Laura R. Saslow, Patricia J. Moran, Sarah Kim, Priyanka K. Wali, Hiba Abousleiman, Robert Richler, Samantha Schleicher, Veronica M. Goldman, Alison Hartman, Cindy Leung, Wendy Hartogensis, Frederick M. Hecht

**Affiliations:** 1 UCSF Department of Psychiatry, Center for Health and Community, San Francisco, CA USA; 2 UCSF Osher Center for Integrative Medicine, 1545 Divisadero Street, Suite 301, San Francisco, CA 94115 USA; 3 Department of Health Behavioral and Biological Sciences, The University of Michigan, School of Nursing, Ann Arbor, MI USA; 4 UCSF Division of Endocrinology, Diabetes and Metabolism, Department of Medicine, San Francisco General Hospital, San Francisco, CA USA; 5 University of Maryland, School of Medicine, Annapolis, MD USA; 6 Department of Psychology, Drexel University, College of Arts and Sciences, Philadelphia, PA USA; 7 Department of Nutritional Sciences, University of Michigan, School of Public Health, Ann Arbor, MI USA

**Correction to: Nutr Metab**

**https://doi.org/10.1186/s12986-019-0383-2**

Following publication of the original article [1], the author reported that the co-author’s name was missing in the original article.

1. In the correction article the co-author Priyanka K. Wali is added.
